# Empathy, motivation, and P300 BCI performance

**DOI:** 10.3389/fnhum.2013.00642

**Published:** 2013-10-17

**Authors:** Sonja C. Kleih, Andrea Kübler

**Affiliations:** Department of Psychology, University of WürzburgWürzburg, Germany

**Keywords:** brain–computer interface, motivation, empathy, ERP, P300, psychological variables

## Abstract

Motivation moderately influences brain–computer interface (BCI) performance in healthy subjects when monetary reward is used to manipulate extrinsic motivation. However, the motivation of severely paralyzed patients, who are potentially in need for BCI, could mainly be internal and thus, an intrinsic motivator may be more powerful. Also healthy subjects who participate in BCI studies could be internally motivated as they may wish to contribute to research and thus extrinsic motivation by monetary reward would be less important than the content of the study. In this respect, motivation could be defined as “motivation-to-help.” The aim of this study was to investigate, whether subjects with high motivation for helping and who are highly empathic would perform better with a BCI controlled by event-related potentials (P300-BCI). We included *N* = 20 healthy young participants naïve to BCI and grouped them according to their motivation for participating in a BCI study in a low and highly motivated group. Motivation was further manipulated with interesting or boring presentations about BCI and the possibility to help patients. Motivation for helping did neither influence BCI performance nor the P300 amplitude. Post hoc, subjects were re-grouped according to their ability for perspective taking. We found significantly higher P300 amplitudes on parietal electrodes in participants with a low ability for perspective taking and therefore, lower empathy, as compared to participants with higher empathy. The lack of an effect of motivation on BCI performance contradicts previous findings and thus, requires further investigation. We speculate that subjects with higher empathy who are good perspective takers with regards to patients in potential need of BCI, may be more emotionally involved and therefore, less able to allocate attention on the BCI task at hand.

## INTRODUCTION

One goal of brain–computer interface (BCI) research is to support people with severe motor impairment who need assisted communication (Kübler and Müller, 2007). Event-related potentials (ERPs) are amongst the most efficacious input signals for BCI (Sellers et al., 2012), which are elicited after presentation of a rare stimulus (oddball) in a stream of frequent non-target stimuli (Sutton et al., 1965). In such an ERP-based BCI the user has to focus attention on the target stimulus presented either in a row or a column of a character matrix (Farwell and Donchin, 1988). In the classic P300-BCI, all rows and columns of a letter or item matrix are flashed in random order (one sequence), therefore a target character is only flashed twice in one sequence (once in the row and once in the column) while the non-target characters are flashed several times. The target character constitutes an oddball which elicits ERPs, mainly a positive potential 300 ms after stimulus presentation (P300; Sutton et al., 1965). Attention allocation increases the P300 amplitude which is also influenced by the value of the target stimulus (Johnson, 1986).

Even though BCI researchers often claim to aim at providing assistive communication for people with severe motor impairment, BCI paradigms are usually tested with healthy volunteers. This is mostly because prior to involving patients in need who are difficult to reach and with who measurements are time and cost intensive, paradigms should be running flawlessly and possibly need to be improved after first experiences with healthy participants. For some healthy volunteers the knowledge about the goals of BCI research might be a more important reason for study participation than the offered monetary compensation. Those subjects may feel enthusiastic about contributing to further developments in BCI that could one day support communication in people with severe impairment. For others, monetary reward may be the main incentive for study participation. Thus, motivation may differ between these groups of potential study participants which in turn could have an effect on BCI performance.

It has been repeatedly shown that motivation affected BCI performance in healthy participants (Nijboer et al., 2008a,b; Kleih et al., 2011a,b; Käthner et al., 2013) and in severely paralyzed end-users (Nijboer et al., 2010). Manipulating motivation confirmed the proposed effect in healthy participants (Kleih et al., 2010) and end-users (Kleih and Kübler, in press). To date, in the context of P300-based BCI, motivation was manipulated with monetary reward (Kleih et al., 2010; Kleih and Kübler, in press) and it was shown that not the monetary reward had the strongest effect, but the motivation independent of such reward. As healthy subjects could earn at the most 50 Eurocents per correctly selected letter and subjects with motoneurone disease a gift certificate worth 20 Euro if they performed better in a second P300-BCI block than in the first, monetary rewards were small and thus, possibly not sufficiently salient. Both rewards might have been perceived as too low to increase motivation. Further, the effect of monetary reward on the P300 ERP may be weak. Consequently, in this paper we investigated whether a non-monetary motivation manipulation would affect the P300 amplitude and spelling success in a P300-based BCI.

To do so, we first sorted the participants in two groups according to their “motivation for helping” in a high and low motivated group. The motivation of the “highly motivated” group may be interpreted as “intrinsic” motivation (Deci and Ryan, 1985; Ryan and Deci, 2000). However, to be purely intrinsic, the reasons for study participation would have to be solely joy or altruism (Fehr and Fischbacher, 2003) which could both lead to a flow experience (Csikszentmihalyi and Csikszentmihalyi, 1992; Csikszentmihalyi and Charpentier, 2008). As soon as an action is performed because it leads to another goal, this action motivation would be categorized “extrinsic” because an action is not initiated by the action itself but by the consequences resulting from that action (Deci and Ryan, 1985; Ryan and Deci, 2000). Consequently, also for people who participate in BCI studies not only for monetary compensation or reward but also because they would like to support further development of assistive technology, motivation would be second goal extrinsic because severely disabled people may benefit in the future.

**FIGURE 1:**
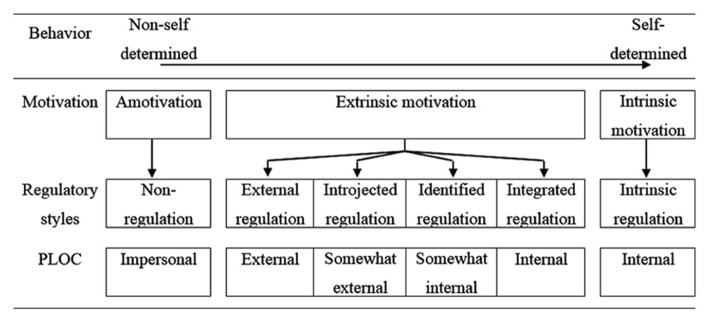
**The self-determination theory (slightly modified from Ryan and Deci, 2000)**. PLOC, perceived locus of control.

In motivation theory, participating in a study because one would like to contribute to research while also being interested in the monetary compensation translates to “introjected” or “identified regulation” as defined by the self determination theory (SDT; Ryan and Deci, 2000; see **Figure 1**). The SDT states that behavior ranges on a continuum from being completely non-self-determined to being entirely self-determined (see **Figure 1**). Perceived motivation, regulatory styles, and the perceived locus of control (PLOC) also differ according to the level of behavioral self-determination (see **Figure 1**). In the SDT, motivation was classified as either “amotivation” which is no motivation for action initiation, therefore constituting the extreme end of the continuum (no behavior). Or motivation is extrinsic leading to behavior that is self-determined to varying extent. Motivation may finally also be intrinsic which constitutes the other extreme end of the continuum at which behavior is entirely self-determined (see **Figure 1**). Therefore motivation determines whether behavior is executed. If behavior is initiated, the question remains, to which extent a person needs regulatory processes to be persistent in a behavior (regulatory styles) and to which extent a person feels autonomous to show the behavior, i.e., the PLOC.

Amotivation does not lead to any behavior and therefore no regulatory processes are required and the locus of control is perceived to be completely impersonal. Extrinsically motivated behavior is shown as a consequence of “external regulation,” for example, when obeying a command. Behavior due to “introjected regulation” is initiated because it is expected by others or to avoid feelings of guilt or anxiety. In a BCI context this translates to healthy participants who feel obliged to spend their time on BCI experiments because they receive course credits or monetary compensation. “Identified regulation” depends on personal importance or even conscious valuing of a particular behavior; here, PLOC starts to adopt internal aspects (**Figure 1**). In a BCI context, identified regulation would refer to people who participate because it is personally important for them, but still it is not initiated self-determined, but due to external cues (advertisement for participation in the experiment). Finally, “integrated regulation” is characterized by regulatory processes that are congruent with the beliefs of this particular person and therefore represent this person’s way of thinking and acting and behavior initiation is perceived as being an internal process (internal PLOC). The highest level of self-determination is reached when motivation and regulation both are intrinsic and behavior is initiated out of interest, enjoyment and inherent satisfaction with the task.

Empathy is another construct which may influence motivation. It was defined as an affective response resulting from the understanding of another person’s emotional state and is similar to what the other person is believed to feel (Eisenberg and Fabes, 1991). It seems to be possible that healthy students who are interested in BCI experiments feel empathy for the potential BCI end-users, provided perspective taking (Eisenberg and Fabes, 1990). Perspective taking is a prerequisite for empathy (Spinrad et al., 2006). Feelings of empathetic concern with the goal to improve another person’s situation can lead to motivation for pro-social behavior (Penner et al., 2005). In a BCI context, perspective taking could lead to participating in a BCI experiment as this supports BCI research which in turn benefits the end-user. Consequently, participation in a BCI experiment could be interpreted as pro-social behavior. We thus, speculated that empathy could support a shift toward integrated regulation and higher motivation.

On the basis of the SDT it seems further conceivable that there are participants for BCI studies with diverging motivation and regulatory styles. The aim of our study was to investigate whether people who initiate action as a result of integrated regulation would achieve better performance in a BCI task than those with introjected regulation. We defined performance as accuracy which corresponds to the percentage of correctly selected letters. We hypothesized (hypothesis H1) that we could strengthen or weaken participants’ pre-existing helping motivation (high or low) by exposing them to a presentation about BCI research in a style that is congruent with initial motivation (very motivated = very informative and lively presentation vs. unmotivated = boring presentation). We also assessed mood because situational mood can change a person’s momentary willingness for helping others (Carlson et al., 1988). We assumed (hypothesis H2) mood to be positively influenced by increasing motivation and negatively by decreasing motivation. We further predicted that highly motivated participants would be better able to focus their attention in a task that could possibly be of benefit for people with disabilities. This possible benefit might increase the value of the target stimulus thereby increasing the P300 amplitude (Johnson, 1986). We hypothesized that motivated participants would present higher P300 amplitudes as compared to less motivated participants (hypothesis H3). Finally, we previously speculated that in a more difficult task the effect of motivation would be more pronounced (Kleih et al., 2010). For this reason we not only decreased stimulus repetitions, but also increased task difficulty by introducing a memory task during spelling which required additional attention. We hypothesized (hypothesis H4) better BCI task performance (in terms of accuracy and speed) and a better performance in the memory task (hypothesis H5) in highly motivated as compared to less motivated participants.

## MATERIALS AND METHODS

### PARTICIPANTS

Participants were *N* = 21 healthy students from the University of Würzburg. We had to exclude one student because he answered socially desirable in all items taken from the SES-17 (Stöber, 1999; see Materials and Methods). The remaining sample consisted of *N* = 20 participants, of who *N* = 14 were female. The average age was *M* = 23.35 (SD = 3.87, range 18–35 years). None of the participants reported a history of neurological or psychiatric disorder. Participants were paid 8 Euro per hour and all were naïve with regards to BCI training. Participants gave informed consent for the study, which had been reviewed and approved by the Ethical Review Board of the Medical Faculty of the University of Würzburg, Germany.

### MATERIALS

Advertisement for the study pronounced the purpose of developing assistive technology for severely ill people. A custom-made questionnaire was designed and inquired participants’ motivation for participation and attitude toward BCI end-users (see **Table 1** for all statements). We assumed that participants who are more extrinsically motivated would report to be more interested in monetary reward (see, for example, items 6, 9, or 12) while participants who were also interested in the task and the BCI users would indicate strong interest and be not only extrinsically motivated (for example, items 4, 13, or 16). We assumed that primarily extrinsically motivated participants would be driven by what Ryan and Deci (2000) called introjected regulation while the also intrinsically motivated group would be more guided by integrated regulation. All statements were rated on a seven-point Likert scale (1 = I strongly disagree, 7 = I fully agree), negative items were inversed, and the sum of all items constituted the total score. Participants re-sent the questionnaire via e-mail and data were median split according to the total score.

**Table 1:** Items of the custom-made questionnaire to separate highly motivated from less motivated participants.

Item no.	Item
1	I would enjoy doing a BCI task
2	I am eager to do my best
3	I believe I can handle the difficulty of the BCI task
4	I would not need a reward as I am very excited about how a BCI works
5	I would like to support and contribute to BCI research
6	I mainly participate in this study because of the monetary reward
7	I am scared that I could blame myself
8	I believe that everybody can control their brain activity
9	I was attracted to this study because of the promised payment
10	When I think about the BCI task I am a little nervous
11	Imagining people being able to communicate using BCIs is very exciting
12	If I did not participate in this study there would be negative consequences for me or I would feel guilty
13	I mainly volunteered for participation in this study because I would like to help people with paralysis
14	This study is a big challenge for me
15	I am knowledgeable in the field of BCI
16	BCI technology can be used to allow people with paralysis for communication. Therefore my main goal in participation is to contribute my share to facilitate these peoples’ lives as their fate really touches me
17	I already used a BCI system
18	I am looking forward to participating

To quantify motivation the Questionnaire for Current Motivation (QCM)-BCI and a visual analog scale (VAS) motivation were used. The QCM-BCI is an adapted version (Nijboer et al., 2008a) of the original QCM (Rheinberg et al., 2001). It comprises 18 items on four motivation components “mastery confidence,” “incompetence fear,” “challenge,” and “interest” that have to be rated on a 7-point Likert scale. On the VAS motivation, participants had to indicate their motivation on a 10 cm long line ranging from 0 (not motivated at all) to 10 (extremely motivated).

We also included a questionnaire to measure empathy as it was hypothesized that people who are highly empathetic would score higher in helping motivation as they could better anticipate the needs of the BCI target population and take over more readily the patients’ perspective. To assess empathy, the Saarbrücker Personality Questionnaire (SPF; Paulus, 2009) was used which is based on the Interpersonal Reactivity Index (IRI) by Davis (1983). It comprises 16 items to be rated on a 5-point Likert scale ranging from 1 (“does not describe me well”) to 5 (“describes me very well”). Four subscales comprise four items each: “perspective taking” (PT), “fantasy” (FS), “empathic concern” (EC), and “personal distress” (PD). The Perspective taking subscale is a measure which indicates how well a person is able to take another person’s point of view (e.g., “I try to look at everybody’s side of a disagreement before I make a decision”) while “fantasy” provides information about a person’s ability to immerse into characters in movies or books (e.g., “I really get involved with the feelings of the characters in a novel”). The Empathetic Concern scale measures how much a person is concerned about another person’s wellbeing (e.g., “I often have tender, concerned feelings for people less fortunate than me”). “Personal distress” measures whether a person feels tense or anxious when in close interaction with other people (e.g., “In emergency situations, I feel apprehensive and ill-at-ease”). While the subscales EC, FS, and PD are considered emotional factors of empathy, PT is considered a cognitive component (Paulus, 2009). Raw values of all four scales were transformed to T-norms.

Another measure used as an indicator for empathy was the “agreeableness” scale (e.g., “I sympathize with others’ feelings”) from the NEO-Five-Factor Inventory (NEO-FFI; Costa and McCrae, 1992; German version: Borkenau and Ostendorf, 1991). “Agreeableness” measures how important it is for a person to get along well with others and to be supportive of others. Items have to be answered on a 5-point Likert scale ranging from 1 (“strong disagreement”) to 5 (“strong agreement”).

The ability to concentrate, and thus, allocate attention on a task was measured with the *d2 test* (Brickenkamp, 1994). The amount of stimuli a person processes is an indicator of “speed,” the amount of errors an indicator of “diligence.” “Performance” included both, speed and diligence by subtracting the sum of all false responses from the amount of all processed stimuli.

As participants could be tempted to answer questionnaires which target empathy and agreeableness socially desirable, we included five items from the social desirability scale (Soziale Erwünschtheitsskala 17, SES-17; Stöber, 1999; e.g., “When in an argument I always stay factual and objective”). These items were integrated into the SPF questionnaire, but analyzed separately.

For the measurement of potential mood changes, we used the German version of the Positive and Negative Affect Schedule (PANAS; Watson et al., 1988; German version: Krohne et al., 1996). The PANAS is subdivided into a positive (PA) and a negative (NA) affect scale, each comprising 10 items which have to be answered on a scale ranging from 1 (“very slightly/not at all”) to 5 (“extremely”). If participants agree more often to positive than negative items it is assumed that this person feels energetic, engaged, and focused while high scores on the negative scale indicate a state of distress and displeasing engagement with others.

To measure the amount of depressive symptoms as a further indicator of mood, we used the short version of the German version of the Center for Epidemiological Studies Depression Scale – CES-D (Allgemeine Depressionsskala = ADS, Hautzinger and Bailer, 1993). The ADS short version (ADS-K) comprises 15 items to be judged on a 4-point Likert scale ranging from 0 (“rarely or none of the time”) to 3 (“most or all of the time”) (for example, “I had trouble keeping my mind on what I was doing”). A score above 23 indicates clinically relevant symptoms of depression.

For assessment of memory and to increase task difficulty we used a slightly changed version of the Visueller und Verbaler Merkfähigkeitstest (= Visual and Verbal Memory Test = VVM; Schächtele and Schellig, 2009). The VVM is a test for the assessment of short- and long-term memory performance and therefore also an indicator of attention. Usually, participants have to memorize visual and verbal information in written form and are asked to reproduce what they had learned. We only used the verbal part which was presented acoustically by a native speaker because visual attention had to be focused on the letter matrix. The text “theater” was recorded with a TBone SC 400 microphone and after noise removal, saved as a.wav file which was later used for presentation. Participants had to listen to the 109 word text and were asked to remember as much information as possible while they were copy-spelling with the P300-BCI. We used this test as a measure of the ability to distribute cognitive resources between two tasks (BCI task and memory task). Memory was assessed twice: firstly in free recall participants had to note all the information they remembered and secondly, in cued recall they had to answer unambiguous questions about the text (e.g., “How many seats were in the theater?” or “How expensive was the theater?”). The VVM was evaluated according the test manual with correct responses being rated with up to two points per answer and a maximum of 24 points.

After finalization of the BCI task, participants received a custom-made post measurement questionnaire. We assessed, for example, interest in the BCI information presentation (e.g., “How interested were you in the BCI information session?”) and whether participants were concentrated or felt exhausted by the BCI task (e.g., “Did you feel exhausted while using the BCI and listening to the memory task?,” “Did you primarily focus on the BCI task or the memory task?” or “What else were you thinking about?”).

### STIMULI AND PROCEDURE

The motivated (*N* = 9; MG) and unmotivated group (*N* = 11, UG) were separated by median split (MD = 4.17) and did not differ concerning gender (*N* = 3 males in both groups) and age (*M*_MG_ = 23.22, SD = 5.21; *M*_UG_ = 23.45, SD = 2.56). Groups were invited for an information presentation about BCI scheduled 1 week before the BCI task. Prior to the presentation participants filled in questionnaires about demographic data, the VAS, QCM-BCI, SPF (including the SES-17), and NEO-FFI agreeableness scale. Then both groups listened to one of two 25 min presentations. To further increase the motivation in the MG, the presentation was designed following instructional design criteria (Mayer, 2003; Reigeluth, 2009) by supporting text information with relevant other media such as pictures and videos which showed the BCI used by severely paralyzed end-users. The presenter spoke in conversational style and reported experiences with patients and stressed how important volunteers are for further development of BCI by giving examples and demonstrations of the interaction between the BCI expert and the end-user. The audience was invited to ask questions at any time. To decrease motivation in the UG, instructional design criteria were ignored and the presentation was restricted to black and white power point slides. Either no additional material such as pictures for illustration was offered or it was not congruent with the text information. No examples to clarify the content were provided and no videos were shown. Text was read from notes with a monotonous voice in ex-cathedra style. While the motivating presentation focused on the importance of BCI research for potential end-users, the demotivating presentation explained only the very basics of EEG recording such as what an electrode looks like and what material it is usually made of; the targeted end-users were mentioned only briefly.

After the presentation, attention was assessed with the d2 and motivation again with the VAS (see **Table 2**). An appointment for the BCI session was scheduled with every participant for the week after the presentation.

**Table 2:** Separation of participants into the motivated group (MG) and the unmotivated group (UG) and questionnaires that were assessed in both groups.

*N* = 20
**t1 group distribution one week prior to information session:**
custom-made questionnaire to split groups for the information presentation
*N* = 9 motivated group (MG)	*N* = 11 unmotivated group (UG)
**t2 prior to information session:**
VAS, QCM-BCI, SPF, SES-17, NEO-FFI agreeableness
Motivating presentation	Demotivating presentation
**t3 after information session:**
d2, VAS
**t4: prior to BCI task**
VAS, PANAS, QCM-BCI, ADS-K
**Calibration**
**Copy-spelling**
2 blocks, each comprising 4 runs (both words twice) VVM presentation: run 1 or 5
**t5: after BCI task**
VAS, QCM-BCI, PANAS, custom made post measurement questionnaire

During the second appointment, the BCI session, participants were first asked to fill in the VAS, PANAS, QCM-BCI, and ADS-K (see **Table 2**). Then participants received an instruction on how to use the P300-BCI and a written information sheet in which the main contents of the presentation they had attended the week before were summarized to reactivate their motivational state before the P300-BCI spelling task.

For the spelling task, participants were presented with a 6 × 6 matrix which contained the German alphabet and numerals 0–9. Participants first completed two copy-spelling (Kübler et al., 2001) calibration runs in which the words “BRAIN” and “POWER” had to be spelled to derive classification coefficients for the following eight experimental copy-spelling runs. The word-to-copy appeared above the matrix and next to it the letter to be copied (target character) in parenthesis. The participants’ task was to pay attention to the target character and to silently count the number of times it was intensified. For each target character three sequences of flashes (one trial) were presented. As the number of sequences was low, no dynamic stopping method (Schreuder et al., 2013) was applied. Each flash lasted 31.25 ms followed by an inter-stimulus interval of 350 ms. After one trial the matrix stopped flashing for 5 s in which the participant had time to locate the next target character to be spelled. After calibration, participants performed two blocks of copy-spelling, each comprising four runs. In one run five characters had to be spelled (“BLUME” or “RADIO”). Thus, in each block both words had to be spelled twice. In none of the copy-spelling runs feedback was provided to avoid motivating the participants by correctly selected letters. The auditory VVM memory task was presented to the participants with a Sony MDR-15 headphone. Presentation of the memory task was counterbalanced across all subjects to control for fatigue. The text to memorize was thus, presented either during run 1 or during run 5. While cued recall was required directly after the presentation, free recall was obtained 20 min later either after run 4 or after run 8, respectively. After spelling, participants again filled in the VAS, PANAS, QCM-BCI, and the custom-made post-measurement questionnaire. Therefore, all motivation and mood questionnaires were assessed three times with exception of the VAS motivation and mood which were assessed four times (see **Table 2**). All participants were offered the opportunity to spell one or two additional words in the free-spelling mode of the system.

### DATA ACQUISITION AND CLASSIFICATION

EEG data collection was controlled by the BCI2000 (Schalk et al., 2004). The electroencephalography (EEG) was measured with an electrode cap (easy cap) with 12 Ag/AgCl electrodes located at positions F3, Fz, F4, C3, Cz, C4, P3, Pz, P4, PO7, PO8, and Oz following the international 10–20 standard system (Sharbrough et al., 1991) referenced to the right and grounded to the left mastoid. Four electrooculography (EOG) electrodes were placed at the temples on a point between the hairline and the eye (left and right, horizontal EOG) and above and below the left eye in line with the pupil when looking straight (vertical EOG). Data was filtered online with a high pass of 0.1 Hz, a low pass of 30 Hz and a notch filter at 50 Hz. The EEG signal was amplified using a g.USBamp (Guger Technologies, Austria). Impedance was kept below 5 kΩ and the sampling rate was 256 Hz. Data were processed and stored on a laptop. For data classification online and offline stepwise linear discriminant analysis (SWLDA) was applied. SWLDA separates the data into two classes (target and non-target signals) both obtaining equal covariance matrices. It calculates a linear equation which depends on the spatiotemporal features of the signals and separates the data classes. This maximizes the distance between the means of the two classes while also minimizing the variance within one class (Lotte et al., 2007). Input features that predict the target label statistically significant are added to the linear equation to explain the largest amount of unique variance while those which are no longer significant are removed. Further details on SWLDA are described, for example, in Krusienski et al. (2008).

### DATA ANALYSIS

For all questionnaire data, scores were transformed into the according norms for further statistical analysis. The EEG data were corrected for artifacts (>50 μV) and baseline (-100 to 0 ms). The P300 was defined as the maximum positive peak occurring between 200 and 600 ms after stimulus onset and was chosen by semiautomatic global peak detection of Brain Vision Analyzer 2^®^ (Brain Products, Germany). Targets and non-targets were averaged and grand averages were compared for the two motivation groups. Spelling speed was measured as the number of sequences needed offline to achieve 70% accuracy. For statistical analysis, cases were weighted as the number of participants was unequal in the two groups. We used IBM SPSS 20^®^ as analysis software. Dependent variables were mood, empathy scores, P300 amplitudes, and BCI performance (online accuracy), spelling speed and performance in the memory task. The level of significance was set to α = 0.05.

## RESULTS

If not indicated otherwise, we used repeated measures ANOVA with time of measurement as within subjects’ factor and group (MG and UG) as between-subjects factor. Time of measurement either comprised three levels, before the information presentation, and before and after the BCI session or four levels (VAS scales); before and after the information session and before and after the BCI session.

### MOTIVATION

To investigate the effect of motivation manipulation and motivation during the BCI task, we used the VAS motivation and the QCM-BCI. For the VAS we found a main effect of *group* for overall motivation [*F*_(1,18)_ = 5.34,* p *< 0.05].**The MG (*M *= 7.84, SD**= 1.81) was significantly more motivated than the UG (*M *= 6.43, SD**= 1.35) confirming successful grouping and sustained higher motivation in MG as compared to UG [*F*_(1,18)_ = 5.34,* p *< 0.05]. For the QCM-BCI we found a significant main effect of *group* for *interest* with the motivated group being significantly more interested (*M* = 5.12, SD = 1.04) than the unmotivated group [*M* = 4.13, SD = 0.72; *F*_(2,36)_ = 8.98, *p* < 0.01], which again confirmed successful grouping. We did not find a main effect of time. Thus, we reject H1 which stated that the motivational state (motivated vs. unmotivated) prior to the information session could be further intensified by our manipulation procedure.

### MOOD

To investigate the effect of motivation manipulation on mood and mood during the BCI task we used the VAS mood and the PANAS. To control for a possible bias caused by pre-existing depressive symptoms, we used the ADS-K. The VAS mood yielded a significant main effect of *group* [*F*_(1,18)_ = 15.08, *p* < 0.01] with MG being in significantly better mood (*M* = 7.71, SD = 1.19) compared to UG (*M* = 6.19, SD = 1.37). In the PANAS a significant main effect of *time* was found [*F*_(1,18)_ = 21.28, *p* < 0.001] with higher negative affect scores before the BCI measurement (*M* = 14.05, SD = 2.93) as compared to thereafter (*M* = 11.30, SD = 1.96). Concerning the ADS-K scores no signs of depression and no group differences were found. All other effects were not significant.

Therefore, H2 of better mood when being motivated was only partially supported by the data because the motivated group was in better mood as indicated by VAS, but this difference existed from the beginning independent of motivation manipulation. The decrease of negative affect was independent of group.

### EMPATHY

Regarding empathy we used multivariate analysis of variance (MANOVA) with the SPF subscale values and the NEO-FFI agreeableness scale. No significant results were found.

### ATTENTION AND P300 AMPLITUDES

As an indicator of attention we used the d2 scores and the P300 amplitudes in the BCI task. Two participants of the MG did not understand the d2 instruction and therefore crossed out the wrong letters throughout the whole test. Thus, for d2 analyses only data of *N* = 18 participants were available. H3 stated that highly motivated participants would better allocate attention leading to higher P300 amplitude and better performance. MANOVA with d2 subscales as dependent variables, revealed significantly higher *concentration performance* in the UG [*F*_(1,16)_ = 10.10, *p *< 0.01, *M*_UG_ = 93.36 SD_UG_ = 5.68 vs. *M*_MG_ = 71.00, SD_MG_ = 22.46]. There was a trend for the UG to be more *diligent* [*F*_(1,16)_ = 4.50, *p *= 0.05, *M*_UG_ = 72.00 SD_UG_ = 25.74 vs. *M*_MG_ = 42.86 SD_MG_ = 32.40]. *Speed* did not differ between groups.

P300 amplitudes at Pz were on average *M* = 7.32 μV (SD = 2.53) in the MG and *M* = 7.10 μV (SD = 1.78) in the UG. When entering electrode position (Fz, P3, Pz, P4, C3, Cz, C4) as within-subjects variable in the repeated measures ANOVA a significant main effect for *electrode* [*F*_(2.41,43.31)_ = 15.39, *p* < 0.001, after Mauchly’s test of sphericity was significant χ^2^_(20)_ = 64.31, *p* < 0.001, Greenhouse–Geisser corrected (ε = 0.49)] but no effect of *group* was found.

Therefore, H3 of better attention allocation as indicated by higher d2 test performance and higher P300 amplitudes in the MG had to be rejected. Contradictory to H3 we found that the UG concentrated better and was more diligent (trend) than the MG.

### PERFORMANCE AND SPELLING SPEED

H4 postulated better BCI performance measured as online accuracy and faster spelling speed measured as sequences needed for correctly spelling 70% accuracy with one, two, and three sequences in the MG compared to the UG. Overall, participants achieved an average online accuracy of *M* = 97.15% (SD = 2.78). The MG reached an accuracy of *M* = 97.00% (SD = 2.68) and the UG an accuracy of *M* = 97.27% (SD = 2.99). The average single trial accuracy (offline) reached 76% or above in both groups. A 4 × 2 repeated measures ANOVA with *sequences* (after one, two, three sequences offline, and overall online) as within-subjects factor and group as between-subjects revealed a main effect of *sequences* [*F*_(1.99,35.86)_ = 34.69, *p* < 0.00, Greenhouse–Geisser corrected (ε = 0.79) after Mauchly’s test for sphericity was significant χ^2^_(5)_ = 29.24, *p* < 0.05]. Within-subjects contrasts were in the expected direction (1 < 2 < 3). No significant differences between groups were found. Thus, H4 of higher performance and spelling speed in the MG had to be rejected.

### SHORT-TERM MEMORY AS AN INDICATOR OF ATTENTION

The fifth hypothesis stated that we could increase task difficulty by adding a memory task to the copy-spelling task and that motivated participants were better in the VVM task and would therefore remember more facts from the memory test in the direct recall and the free recall after 20 min. However, the additional memory task was probably too difficult for participants as most did not reach average T-norm values of between 40 and 60 but ranked much below (overall *M* = 18.15, SD = 14.09 for the cued recall and *M* = 24.05, SD = 11.15 for the free recall). To investigate group differences, we compared the MG and UG and found a significant main effect for *time* [*F*_(1,18)_ = 4.58, *p* < 0.05] but no effect of *group*. Surprisingly, within-subjects contrasts revealed significantly higher memory performance in the free as compared to the cued recall [*F*_(1,18)_ = 8.80, *p* < 0.01]. However, H5 of better memory performance in the MG could not be confirmed by the data.

### FREE SPELLING

To assess another behavioral indicator of motivation, participants were asked after finishing copy-spelling whether they wished to try free-spelling with a self-chosen word. Nineteen participants accepted the offer and only one participant who belonged to the unmotivated group refused to try free-spelling. The freely chosen words were between three and six characters long and there was no group difference with regards to time spent with free-spelling [*t*_(18)_ = 0.90, *p* = 0.11].

### POST-MEASUREMENT QUESTIONNAIRE

Results of the post-measurement questionnaire revealed that from the MG *N* = 7 participants reported the information presentation to be very interesting while only *N* = 3 from the unmotivated group did so. In the UG most participants judged the information presentation as completely uninteresting (*N* = 6). Thus, on the descriptive level we can state that the information presentations were indeed perceived qualitatively different.

Furthermore, *N* = 5 from the MG and *N* = 8 from the UG reported that they subjectively did not judge the BCI task as exhausting. When being asked whether participants were primarily focused on the BCI spelling or the VVM auditory task, *N* = 3 from MG and *N* = 8 from UG reported to have focused primarily on the BCI spelling task. All participants in the MG but only *N* = 3 in the UG reported that they were concentrated during the task.

### POST HOC ANALYSIS FOR EMPATHY

The values in the SPF subscale perspective taking were considered to be highly important in this paradigm as we aimed at specifically increasing “motivation for helping.” Therefore we regrouped the sample by the median of their SPF PT values. All participants who had values of 46.27 (Md) or less (*N* = 10) were grouped into the “less able to take others’ perspective” group (LAPT), all participants who had values above 46.27 (*N* = 10) were grouped into the “highly able to take others’ perspective” group (HAPT). In the HAPT group there were *N* = 4 participants of the original high motivation group and *N* = 6 participants of the original unmotivated group. There were *N* = 4 male participants in this group and mean age was 23.52 (SD = 4.79). In the LAPT group there were therefore *N* = 5 participants of the original high motivation group and *N* = 5 participants of the low motivation group. Two participants were male and mean age was 23.15 (SD = 3.21).

We post hoc applied the hypotheses H1–H5 to the LAPT vs. HAPT groups.

#### Motivation, mood, empathy

For all dependent variables the same analyses were performed as described above. Motivation (VAS, QCM-BCI) and mood (VAS, PANAS, ADS-K) did not differ between the groups.

Regarding empathy, we used MANOVA with the SPF subscale values (other than PT) and the NEO-FFI agreeableness scale as dependent variables and group as between-subjects variable. The HAPT group showed significantly higher *Empathetic Concern* [*F*_(1,18)_ = 5.48, *p *< 0.05] and a trend toward lower *Personal Distress* [*F*_(1,18)_ = 3.43, *p *= 0.08].

#### Attention and P300 amplitude

The ability to concentrate (d2 scores) did not differ between groups. Thus, H3 of higher capability to focus attention in the HAPT had to be rejected.

**FIGURE 2:**
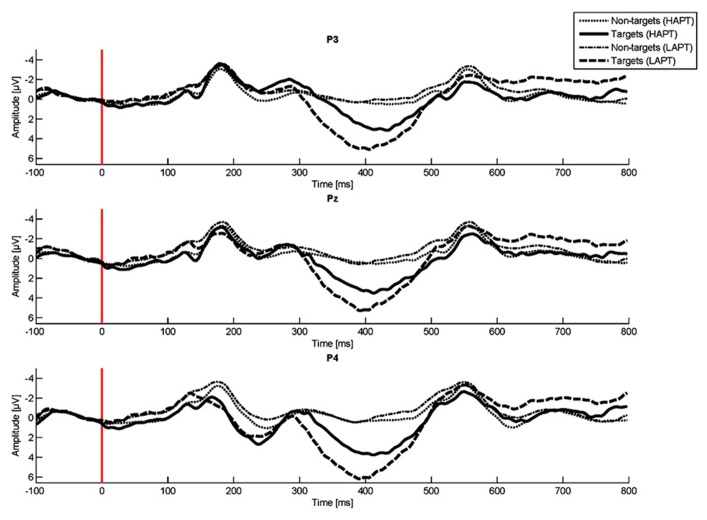
**P300 amplitudes at P3, Pz, and P4 for the low (LAPT) and the high (HAPT) *perspective taking* group**.

The comparison of P300 amplitudes with the within-subjects factor *electrode* (Fz, C3, Cz, C4, P3, Pz, P4) and the between-subjects factor *group* yielded main effects for *electrode* [*F*_(6,108)_ = 13.50, *p* < 0.001] and *group* [*F*_(1,18)_ = 6.64, *p* < 0.05]. Post hoc tests revealed a significantly higher P300 amplitude at P3 (mean difference = 2.03, *p* < 0.05, *M*_LAPT_ = 5.64, SD = 2.29, *M*_HAPT_ = 3.73, SD = 0.96), Pz (mean difference = 2.14, *p* < 0.05, *M*_LAPT_ = 6.90, SD = 2.48, *M*_HAPT_ = 4.87, SD = 1.59) and P4 (mean difference = 1.94, *p* < 0.05, *M*_LAPT_ = 5.83, SD = 2.04, *M*_HAPT_ = 4.02, SD = 0.88) in the LAPT as compared to the HAPT group (see **Figure 2**). Contradictory to H3 of higher P300 amplitudes in the HAPT, we found higher P300 amplitudes in the LAPT group.

#### Performance and spelling speed

Again a 4 × 2 repeated measures ANOVA with *sequences* (after one, two, three sequences, and overall online) as within-subjects factor and *group* as between-subjects factor revealed a main effect of *sequences* [*F*_(2.02,36.43)_ = 38.38, *p* < 0.000, Greenhouse–Geisser corrected (ε = 0.68) after Mauchly’s test for sphericity was significant χ^2^_(5)_ = 26.86, *p* < 0.05]. Within-subjects contrasts were in the expected direction (1 < 2 < 3). For overall online performances, no significant differences between groups were found (*M*_L__APT_ = 98.60, SD = 1.63; *M*_H__APT_ = 95.94, SD = 2.98). Thus, H4 of higher performance and spelling speed in the HAPT as compared to the LAPT group had to be rejected.

## Discussion

In this study we investigated the effect of motivation for helping and perspective taking on BCI performance. We successfully sorted participants in a motivated and unmotivated group, but were not able to increase and decrease the initial motivation by means of a motivation congruent presentation. With our advertisement it seems likely that only participants who were interested in the topic were attracted to learn about the functionality of how a BCI works. Additionally, just the fact that participants received information in the BCI information presentation, irrespective of the patient-related content, might have increased their involvement in the BCI task. Maybe confronting participants with a situation in which empathy could have led to an action directly would have been more successful. Batson et al. (1983) administered mild electrical shocks to a test person who had to perform digit-recall. Observers of this situation indicated high empathy and willingness to help the test person. Dovidio et al. (1990) presented their participants with a video of a student who had been ill and thus could not fulfill all requirements in time for entering graduate school. Participants indicated commitment to support this person actively. In such scenarios, motivation for helping had a clear action direction which probably facilitated action initiation. As we could not confront our subjects with a real BCI end-user, our manipulation may have been less powerful. Furthermore, the time between the presentation and the BCI experiment may have caused possible effects to extenuate. Our motivation manipulation which was supposed to strengthen pre-existing motivation was quantitatively unsuccessful as motivation of the MG could not be increased and that of the UG not decreased; however, qualitatively it was perceived as intended. The initial motivation had no effect on any of our dependent variables and thus, we had to reject H1.

Our second hypothesis stated that we could increase mood together with motivation. As our motivation manipulation failed, we also had to reject H2 even though we found better mood in the highly motivated group. The more negative affect participants experienced before the BCI task as compared to thereafter, was independent of group and therefore may be attributed more to the test situation instead of the motivation manipulation.

Our results were also contradicting our hypothesis H3 which stated that subjects of the MG should present with higher P300 amplitudes due to better allocation of attention. Thus, we could not confirm Kleih et al. (2010) who found higher P300 amplitudes in highly motivated as compared to less motivated participants. Attention allocation as measured with the d2 was higher in the UG as compared to the MG. A possible explanation is that participants in the motivated group were very attentive throughout the presentation and thus, had fewer resources for attention allocation in the d2 test. Similarly, after group re-distribution, participants who were less able to take perspective, i.e., were less empathetic (LAPT group), presented with higher P300 amplitudes. Both results are in line with Johnson’s model which postulates that allocation of attentional resources affects the P300 amplitude (Johnson, 1986). The resources of the motivated and the highly empathetic group might have been limited. Avenanti et al. (2005) showed that empathetic involvement affects brain responses. Their participants watched needles being penetrated through a body model and in consequence participants’ motor evoked potentials (MEPs) as measured with transcranial magnetic stimulation (TMS) decreased in amplitude in sensorimotor regions corresponding to the penetrated body parts. The authors assumed that empathetic involvement led to emotional arousal which had an inhibitory effect on the MEP response. Although we did not measure emotional arousal it might well be that the more subjects were involved in the purpose of BCI the stronger the negative effect on the P300 amplitude. The Yerkes and Dodson (1908) law which postulated an inverted U-shaped relation between task performance and arousal may explain this result. It is important to note that in this study we refer to arousal as a state of emotional involvement and not to general cortical excitability which is also a definition of arousal (Polich and Kok, 1995; Polich, 2007).

Eisenberg and Eggum (2009) explain lower attention capacity in the context of empathy by the lack of self-regulation. They state that in case empathy is not characterized by compassionate concern about a person’s wellbeing but by personal distress, attentional resources are negatively affected and lead to a self-focus. The primary goal of a person would be to alleviate one’s own negative state. In our BCI context, this translates to higher arousal in the highly empathetic group because bearing in mind the health state of possible BCI end-users could have caused personal distress and lower attentional resources for the BCI task. Furthermore, in case self-regulatory processes are required to cope with emotional involvement, also less pro-social behavior is observed (Eisenberg and Fabes, 1990; Penner et al., 2005) as the focus of the person is self-centered. Therefore, participants in our study probably did not take extra effort to concentrate specifically hard on the VVM task as they were focusing on reduction of possible negative emotions.

BCI performance could not be positively influenced, neither by motivation, nor by empathy which rejects our hypothesis H4 as well as previous work by Kleih and Kübler (in press) who found a correlation between mastery confidence and the number of sequences needed to spell with 70% accuracy. However, Kleih and Kübler (in press) investigated a sample of ALS patients who were highly intrinsically motivated and also showed a ceiling effect of performance. This patient sample is not comparable to healthy students who know that for themselves a BCI is not required for communication purposes and for who integrated regulation, i.e., acting in congruence with one’s own beliefs is probably the highest motivation level to reach (Ryan and Deci, 2000).

In the memory task measured with the VVM no significant differences between the motivated and unmotivated groups nor the HAPT and the LAPT groups were found so we had to reject our fifth hypothesis H5. This might be explained by the task difficulty. As it turned out, the memory task was basically ignored by participants. Accuracies were constantly high in the BCI task, even though only three sequences were used while the T-norm scores for the VVM were far below the normal average. This indicates that participants allocated attentional resources on the BCI task only, while ignoring the auditory memory task. As Carr (2004) explained, for every incoming stimulus, it needs to be decided whether this stimulus should be processed early (= superficially) or deeply ( = determination of stimulus identification, meaning, and preparation for possible response; Carr, 2004). In this VVM task, the auditory stimuli were probably not processed deeply as attention was focused on the primary area of stimulus presentation on the computer screen. Consequently no bimodal processing occurred. This phenomenon of impaired processing of auditory stimuli when being presented together with visual stimuli is known as Colavita visual dominance effect (Colavita, 1974). The Colavita effect was repeatedly reported (Laurienti et al., 2004; Molholm et al., 2004; Spence, 2007,^2009^) and was found not to be simply dependent on the intensity or probability of the presented stimuli (Spence et al., 2012). Koppen and Spence (2007) explained the Colavita effect with endogenous attention allocation toward visual perception to compensate for its low alerting properties in comparison to the auditory domain which immediately causes an orientation reaction (Talsma et al., 2007; Spence et al., 2012). Indeed it was shown that manipulation of endogenous attention by, e.g., decreasing the likelihood for the visual stimulus decreased also the Colavita effect (Egeth and Sager, 1977).

With regards to the SDT (Ryan and Deci, 2000), it remains unclear, whether participants applied *introjected* (avoidance of feelings of guilt) or *integrated* regulation. But it might be that participants in the HAPT group were too much involved in the task and their *identified regulation* hindered them to focus on the BCI task. The LAPT group which was more emotionally distant did not show this detrimental effect on P300 amplitude.

In conclusion, we could not increase or decrease state motivation by emphasizing the user-centered aspect of BCI research. Contrary to previous studies, motivation did not affect P300-BCI performance. Subjects with lower trait empathy, who were likely to be less emotionally involved, and thus, better able to focus attention on the task, presented larger P300 amplitudes than those with higher trait empathy. Therefore, we conclude that there is a moderate influence of empathy on the P300 within a BCI paradigm. Further research on empathy and motivation in BCI is required.

## Conflict of Interest Statement

The authors declare that the research was conducted in the absence of any commercial or financial relationships that could be construed as a potential conflict of interest.
